# Correction: Dominant formation of disulfidic linkages in the sulfur cross-linking reaction of isoprene rubber by using zinc stearate as an activator

**DOI:** 10.1039/d5ra90126j

**Published:** 2025-11-07

**Authors:** Yuta Sakaki, Ryota Usami, Atitaya Tohsan, Preeyanuch Junkong, Yuko Ikeda

**Affiliations:** a Graduate School of Science and Technology, Kyoto Institute of Technology Matsugasaki, Sakyo Kyoto 606-8585 Japan; b Department of Materials and Production Technology Engineering, Faculty of Engineering, King Mongkut’s University of Technology North Bangkok Wongsawang, Bangsue Bangkok 10800 Thailand; c Center for Rubber Science and Technology, Kyoto Institute of Technology Matsugasaki, Sakyo Kyoto 606-8585 Japan; d Research Strategy Promotion Center, Kyoto Institute of Technology Matsugasaki, Sakyo Kyoto 606-8585 Japan; e Faculty of Molecular Chemistry and Engineering, Kyoto Institute of Technology Matsugasaki, Sakyo Kyoto 606-8585 Japan yuko@kit.ac.jp

## Abstract

Correction for ‘Dominant formation of disulfidic linkages in the sulfur cross-linking reaction of isoprene rubber by using zinc stearate as an activator’ by Yuta Sakaki *et al.*, *RSC Adv.*, 2018, **8**, 10727–10734, https://doi.org/10.1039/C8RA00544C.

The ORCiD previously listed for Yuko Ikeda was incorrect. The correct ORCiD is: https://orcid.org/0000-0003-2809-8911.

The Royal Society of Chemistry apologises for these errors and any consequent inconvenience to authors and readers.

